# Gut microbiota causally affects cholelithiasis: a two-sample Mendelian randomization study

**DOI:** 10.3389/fcimb.2023.1253447

**Published:** 2023-10-09

**Authors:** Xin Liu, Xingsi Qi, Rongshuang Han, Tao Mao, Zibin Tian

**Affiliations:** Department of Gastroenterology, The Affiliated Hospital of Qingdao University, Qingdao, China

**Keywords:** gallstones, causal relationship, bile salt hydrolase, gut microbiota, Mendelian randomization

## Abstract

**Background:**

The gut microbiota is closely linked to cholesterol metabolism-related diseases such as obesity and cardiovascular diseases. However, whether gut microbiota plays a causal role in cholelithiasis remains unclear.

**Aims:**

This study explored the causal relationship between gut microbiota and cholelithiasis. We hypothesize that the gut microbiota influences cholelithiasis development.

**Methods:**

A two-sample Mendelian randomization method was combined with STRING analysis to test this hypothesis. Summary data on gut microbiota and cholelithiasis were obtained from the MiBioGen (n=13,266) and FinnGen R8 consortia (n=334,367), respectively.

**Results:**

*Clostridium senegalense*, *Coprococcus3*, and *Lentisphaerae* increased the risk of cholelithiasis and expressed more bile salt hydrolases. In contrast, *Holdemania*, *Lachnospiraceae UCG010*, and *Ruminococcaceae NK4A214* weakly expressed bile salt hydrolases and were implied to have a protective effect against cholelithiasis by Mendelian randomization analysis.

**Conclusion:**

Gut microbiota causally influences cholelithiasis and may be related to bile salt hydrolases. This work improves our understanding of cholelithiasis causality to facilitate the development of treatment strategies.

## Introduction

Cholelithiasis (also known as gallstones) is defined as a solid clot in the gallbladder or biliary system (Lammert et al., 2016). Approximately 90% of cholelithiasis occurrences are cholesterol gallstones, while the incidence of other stone types (including black and brown pigment stones) is below 10% (Sun et al., 2022). Approximately 10–20% of the global population has gallstones, and over 20% of cases develop gallstone diseases, such as acute cholecystitis, acute cholangitis, and obstructive jaundice (Cortés et al., 2020). Gallstone disease is one of the most expensive gastrointestinal conditions from a societal perspective (Grigor’eva and Romanova, 2020). Epidemiological studies identified numerous risk factors for cholesterol stones, which include type 2 diabetes, physical inactivity, and over-nutrition (Di Ciaula et al., 2019). This finding can be attributed to the risk factors that lead to excess cholesterol or disruption of cholesterol homeostasis (Rudling et al., 2019).

The gut microbiota has a non-negligible impact on metabolic disorders, including insulin resistance (Gomes et al., 2018), obesity (Abenavoli et al., 2019), and hyperlipidemia (Michels et al., 2022). These disorders are known risk factors for increased hepatic cholesterol synthesis, gallstone formation, and symptomatic gallstones (Di Ciaula et al., 2019). However, the role of intestinal microbiota in gallstone development remains unclear. Elevated levels of cholesterol and bilirubin in the bile and decreased levels of bile salts cause cholesterol gallstones (Sun et al., 2022). Decreased bile salt levels are observed in liver disease and in conditions such as Crohn’s disease or in individuals that have undergone colectomy or intestinal resection, where the enterohepatic circulation of bile salts is impaired (Molina-Molina et al., 2018). These findings led us to hypothesize that the gut microbiota and intestine may play key roles in influencing gallstone formation in the host. The diversity and taxonomy of gut microbiota are associated with bile acid levels in gallstone disease, and increased concentrations of taurodeoxycholic acid and taurocholic acid are associated with the presence of conditionally pathogenic bacteria (Petrov et al., 2020). However, intestinal flora is often influenced by confounding factors, including age, sex, environment, alcohol consumption, diet, and lifestyle (Zmora et al., 2019). Eliminating these confounders in observational studies can be challenging, thereby limiting research on the causal role of gut microbiota in gallstones. Fortunately, Mendelian randomization (MR) analysis can be employed to explore the causal role of intestinal microbiota in the etiology of human diseases independent of confounding factors (O’Donnell et al., 2023).

MR borrows economically inspired statistical techniques to enable researchers to examine the causal factors affecting human diseases (Birney, 2022). Numerous risk factors related to diseases have not established causation owing to the limitations of observational studies that cannot avoid the influence of confounders. Thus, MR has become one of the most effective methods for addressing issues in human biology and epidemiology, including the relationship between intestinal microbiota and disease (Georgakis and Gill, 2021). The correlation between genetic variation and outcome is independent of confounders in MR analysis (Bowden and Holmes, 2019). For instance, MR analysis has revealed that *Bifidobacterium* is causally linked to preeclampsia-eclampsia (Li et al., 2022), and the fecal abundance of *Oscillibacter* and *Alistipes* is causally associated with decreased triglyceride levels (Liu et al., 2022).

This study used summary-level statistics of the genome-wide association study (GWAS) from the MiBioGen and FinnGen consortia to perform a two-sample MR analysis to investigate the causal relationship between intestinal microbiota and cholelithiasis.

## Materials and methods

The two-sample MR study relied on three assumptions to draw conclusions regarding causation. Genetic variants strongly predict microbiome exposure independent of confounding factors and outcomes. Genetic variants influence outcomes through exposure (Emdin et al., 2017).

### Data sources

Single nucleotide polymorphisms (SNPs) related to intestinal microbiota were obtained from the GWAS dataset of the International Consortium MiBioGen and were used as instrumental variables (IVs). This study included 34,024 individuals from 18 cohorts predominantly of European ancestry (including those from the United States, Canada, Israel, South Korea, Germany, Denmark, The Netherlands, Belgium, Sweden, Finland, and the United Kingdom). The dataset provided genotyping data that coordinated 16S ribosomal RNA gene sequencing to examine the relationship between genetic variants and intestinal microbiota by profiling taxonomic classification. A total of 211 taxa were included in the analysis. We selected SNPs that showed significant correlations with genus at a suggestive of genome-wide significance thresholds (P < 1× 10^−5^, F >10) as potential IVs from MiBioGen (Li et al., 2022).

### Data outcome

GWAS summary statistics for cholelithiasis were acquired from the FinnGen Consortium R8 release data. The phenotype “cholelithiasis” was adopted in our research. The GWAS consisted of 32,894 cases and 301,383 controls. The mean age of the patients was 52.11 years old (female: 48.62, male: 59.36). The principal components (sex and age), and the genotyping batch were corrected during the analysis. SNPs were identified with genome-wide significant correlations with cholelithiasis (P < 5 × 10^−8^).

### Instrumental variable filtering

First, SNPs showing significant correlations with the genus were selected at suggestive genome-wide significance thresholds from the MiBioGen as potential IVs (P < 1× 10^−5^, F >10). A chain imbalance threshold (R^2^ < 0.001) and linkage disequilibrium threshold of 10,000 kbp was then applied to ensure independence among the selected SNPs. Single nucleotide polymorphisms that were unclear, duplicated, or palindromic were removed to ensure consistent SNP orientation in the exposure and results. Next, SNPs should have P >1×10^-5^ to ensure that SNPs are independent of the outcome. Finally, the PhenoScanner online tool was used to check whether the SNPs affected the outcome, followed by their manual exclusion. The MR-Egger intercept analysis was employed to test for horizontal pleiotropy (P> 0.05) and leave-one-out analysis was conducted to assess whether each IV affected the overall estimates of the remaining IVs.

### MR analysis

The most frequently used MR methods were used to analyze the causal relationship between intestinal microbiota and cholelithiasis. They were inverse variance weighted (IVW), MR-Egger, weighted median, weighted mode, and simple mode.

### Reverse MR analysis

MR inverse analysis was performed to explore whether cholelithiasis had a causal effect on the identified microbiomes. During this analysis, cholelithiasis and bacteria was considered as an exposure and an outcome, respectively, SNPs with notably correlated with cholelithiasis were taken as IVs.

All data analysis was used R software (version 4.2.1) to conduct. We applied the IVW, weighted median, and MR-Egger regression methods using the R packages of “TwoSampleMR” (version 0.5.6). And “MRPRESSO” package was used to perform the MR-PRESSO analysis.

### SRTING analysis

The STRING database collects and integrates protein-protein interaction (PPI), performs enrichment analysis and highlights proteins in the PPI network (Szklarczyk et al., 2023). We tried to use the STRING to predict the function of BSH in the bacteria (https://string-db.org), and conduct ontology (GO)and KEGG enrichment analysis. Statistical significance was defined as p<0.05.

## Results

### Single nucleotide polymorphism selection

**Figure 1:**
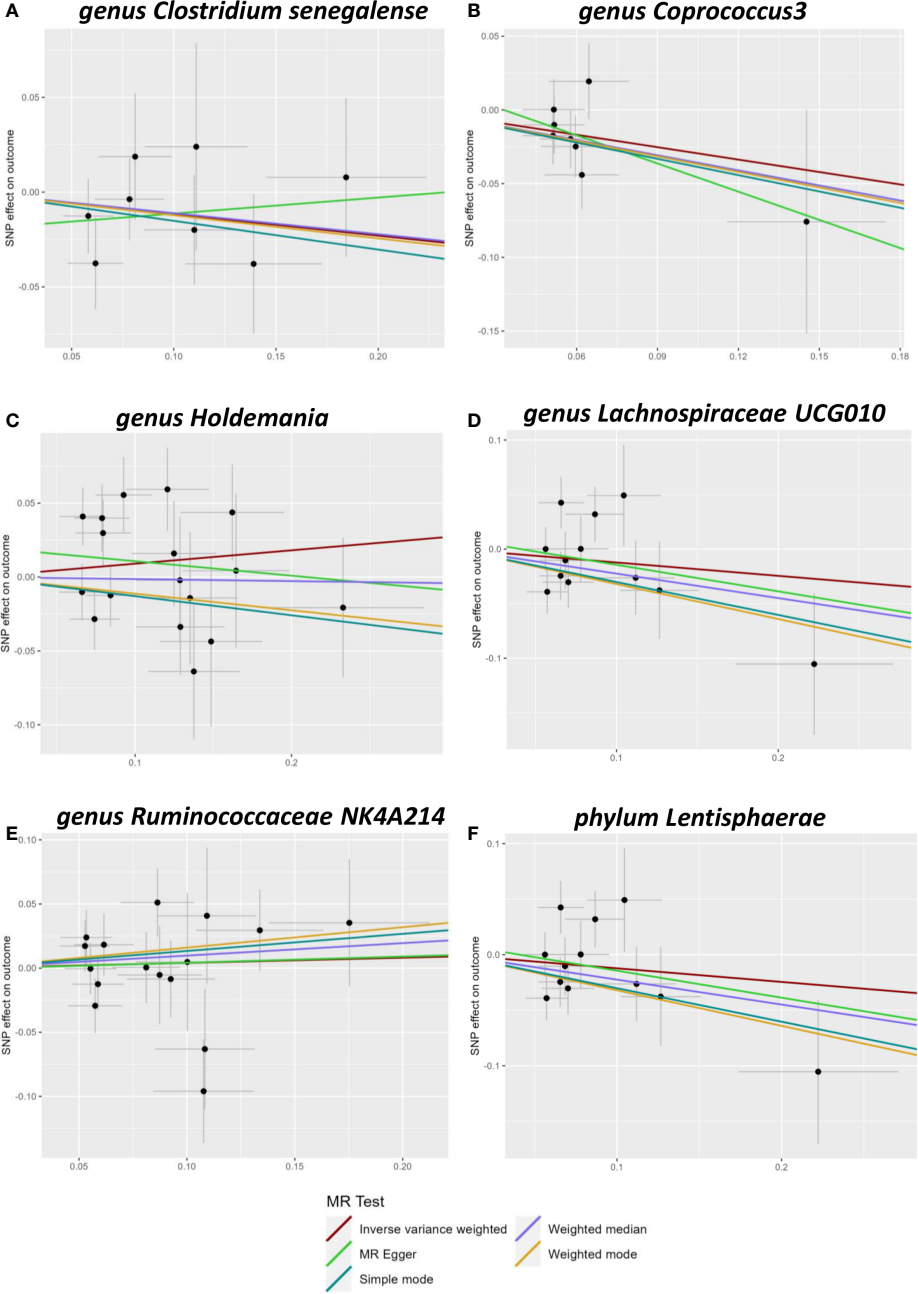
Scatter plots of Mendelian randomization (MR) analysis. The scatter plot of *Clostridiumsenegalense* **(A)**, *Coprococcus3* **(B)**, *Holdemania* **(C)**, *Lachnospiraceae UCG010* **(D)**, *Ruminococcaceae 617 NK4A214* **(E)**, and Lentisphaerae **(F)**.

A total of 2557 SNPs related to 211 taxa were identified as IVs of the gut microbiota. A series of quality control steps was performed resulting in the selection of 72 SNPs associated with six genera and one phylum (based on the IVW P-value < 0.05). Analysis of these 72 SNPs using PhenoScanner showed that no SNPs were related to confounding factors. Heterogeneity between the two samples was tested using Cochran’s Q statistics and no evidence of heterogeneity (p > 0.05) or horizontal pleiotropy of the IVs was observed (MRPRESSO-global, p > 0.05; MR-Egger intercept, p > 0.05) (**Table S1**). The MR analysis results are shown in **Figure 1**.

### Detailed MR results

Five genera (*Clostridiumsenegalense*, *Coprococcus3*, *Holdemania*, *Lachnospiraceae UCG010*, and *Ruminococcaceae NK4A214*) and one phylum (Lentisphaerae) of the microbiome were suggested to have causal relationships with cholelithiasis. IVW analysis suggested that *Clostridiumsenegalense* (OR=1.244, 95% CI:1.091–1.419), *Coprococcus3* (OR=1.236, 95% CI: 1.047–1.458), and the Lentisphaerae phylum (OR=1.081, 95% CI: 1.004–1.164) increased the risk of cholelithiasis. Meanwhile, *Holdemania* (OR=0.911, 95% CI: 0.841–0.987), *Lachnospiraceae UCG010* (OR=0.835, 95% CI:0.740–0.942), and *Ruminococcaceae NK4A214* (OR=0.860, 95% CI:0.768–0.963) had a protective effect on cholelithiasis (**Table 1**).

**Table 1:** The MR analysis of causal effects between gut microbiota and cholelithiasis.

Bacterial taxa (exposure)	MR method	No. SNP	*F*-statistic	*OR*	95%*IC*	*p-*value
Genus- *Clostridium* *senegalense*	IVW	8	31.43	1.24	1.09-1.42	0.001
	MR Egger	8		1.33	0.93-1.90	0.169
	Weighted median	8		1.20	1.00-1.44	0.169
	Weighted mode	8		1.19	0.92-1.53	0.225
	Simple mode	8		1.40	1.06-1.86	0.051
genus *Coprococcus3*	IVW	8	27.44	1.24	1.05-1.46	0.012
	MR Egger	8		1.39	0.54-3.60	0.525
	Weighted median	8		1.22	0.97-1.53	0.088
	Weighted mode	8		1.24	0.86-1.79	0.28
	Simple mode	8		1.24	0.86-1.78	0.294
genus *Holdemania*	IVW	17	48.51	0.91	0.84-0.99	0.023
	MR Egger	17		0.99	0.79-1.25	0.226
	Weighted median	17		0.93	0.83-1.04	0.042
	Weighted mode	17		1.00	0.82-1.23	0.209
	Simple mode	17		1.00	0.80-1.23	0.211
genus *Lachnospiraceae UCG010*	IVW	12	36.14	0.84	0.74-0.94	0.003
	MR Egger	12		0.89	0.61-1.30	0.564
	Weighted median	12		0.87	0.73-1.02	0.084
	Weighted mode	12		0.89	0.67-1.18	0.434
	Simple mode	12		0.87	0.65-1.16	0.360
genus *Ruminococcaceae NK4A214*	IVW	16	28.49	0.86	0.77-0.96	0.009
	MR Egger	16		0.19	0.65-1.28	0.604
	Weighted median	16		0.81	0.70-0.94	0.007
	Weighted mode	16		0.82	0.66-1.01	0.080
	Simple mode	16		0.81	0.63-1.04	0.119
phylum *Lentisphaerae*	IVW	11	88.05	1.08	1.00-1.16	0.038
	MR Egger	11		1.02	0.76-1.38	0.882
	Weighted median	11		1.06	0.96-1.17	0.261
	Weighted mode	11		1.07	0.90-1.27	0.461
	Simple mode	11		1.08	0.91-1.27	0.404

OR, odds ratio; CI, confidence interval; IVW, Inverse variance weighted; No., number; SNP, single-nucleotide polymorphism; MR, Mendelian randomization.

### Sensitivity analysis

**Figure 2:**
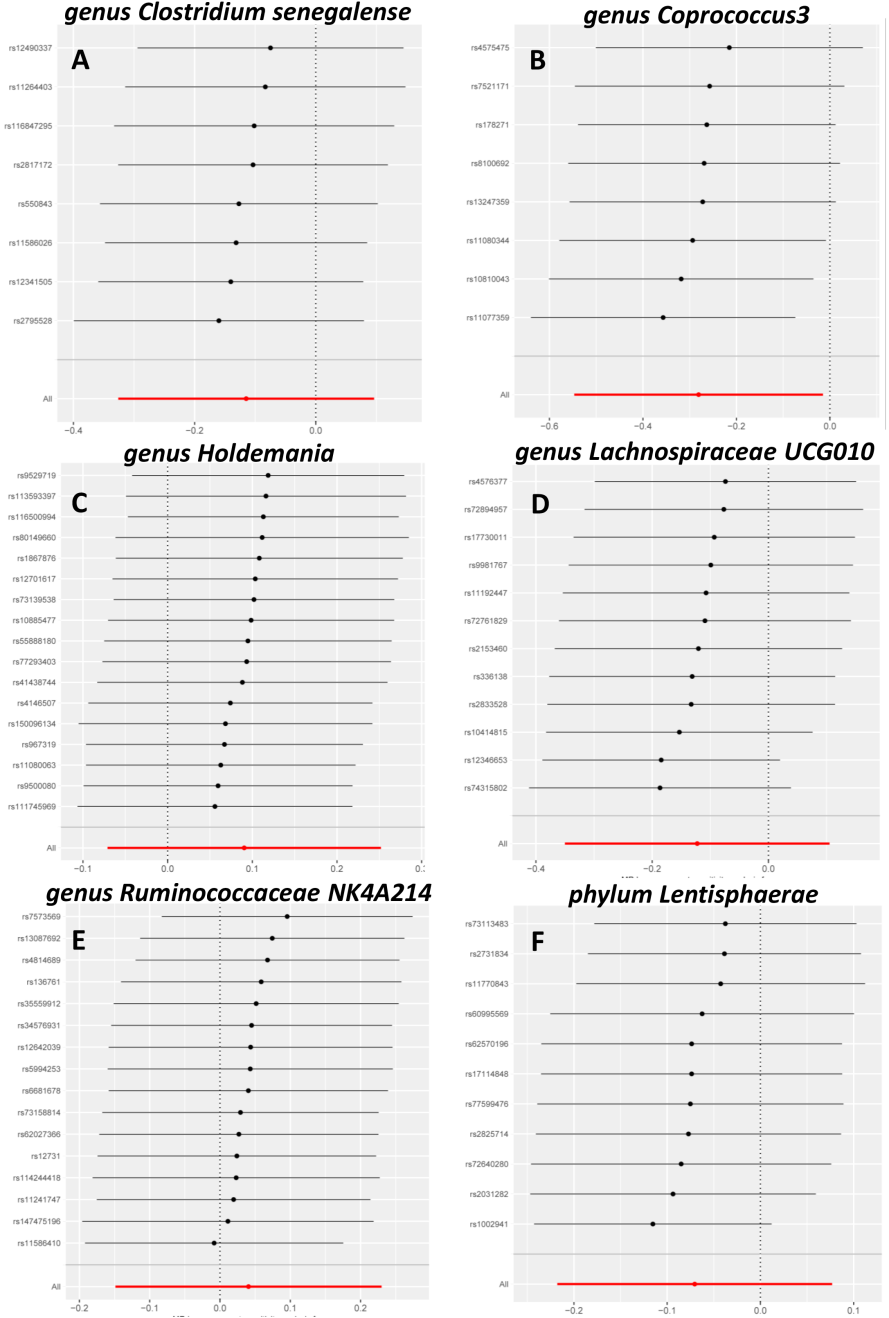
Leave-one-out sensitivity analysis. Leave-one-out sensitivity analysis of *Clostridiumsenegalense* **(A)**, *Coprococcus3* **(B)**, *Holdemania* **(C)**, *Lachnospiraceae* UCG010 **(D)**, *Ruminococcacea*e NK4A214 **(E)**, and *Lentisphaerae* **(F)**.

Sensitivity analysis is necessary to assess the effectiveness of IVWs. The MR-Egger method was used to assess horizontal multi-effectiveness. The MR-Egger intercept and MR-PRESSO global tests indicated a low likelihood of horizontal multiplicity (**Table S1**, p > 0.05). All I2 values in the heterogeneity tests were <50%, and all p-values were >0.05. This finding indicated that our findings were probably not influenced by heterogeneity bias. The MR-Egger intercept and the MR-PRESSO global test showed no significant horizontal pleiotropy. This result indicated that the outliers did not significantly affect the results. Simultaneously, the consistency of the robust IVW (adjusted for the effect of outliers) and MR-PRESSO (adjusted for the effect of horizontal multiplicity) supported the lack of significant outlier effects on the results (**Table S1**). A leave-one-out analysis was also conducted to assess whether each IV affects the overall estimates of the remaining IVs. These results suggested that none of the single IV treatments affected the results (**Figure 2**).

### The causal effects of gut microbiota and cholelithiasis via inverse MR analysis

The reverse causal effects were examined using cholelithiasis as the exposure and intestinal microbiota as the outcome. Forty-three SNPs associated with cholelithiasis were selected as IVs (P < 5 × 10^-8^). Cholelithiasis was causally associated with *Actinobacteria*, *Lachnospiraceae*, *Clostridium innocuum*, *Eggerthella*, *Eubacterium brachy*, *Intestinimonas*, *Paraprevotella*, and *Mollicutes RF9* (**Table 2**). None of the bacteria exhibited a bidirectional causal relationship with cholelithiasis and gut bacteria.

**Table 2:** Inverse MR analysis the causal effects of gut microbiota and cholelithiasis.

Bacterial taxa (outcome)	MR method	No. SNP	*OR*	95%*IC*	*p-*value
Class *Actinobacteria*	IVW	10	1.160	1.059-1.271	0.001
Family *Lachnospiraceae*	IVW	23	0.96	0.929-0.993	0.001
Genus *Clostridium innocuum*	IVW	23	0.930	0.867-0.998	0.043
Genus *Eggerthella*	IVW	23	0.917	0.859-0.978	0.008
Genus *Eubacterium brachy*	IVW	23	0.928	0.865-0.995	0.036
Genus *Paraprevotella*	IVW	23	0.944	0.893-0.999	0.044
Genus *Intestinimonas*	IVW	23	1.048	1.006-1.091	0.026
Order *Mollicutes RF9*	IVW	23	1.048	1.098-1.000	0.048

OR, odds ratio; CI, confidence interval; IVW, Inverse variance weighted; No., number; SNP, single-nucleotide polymorphism; MR, Mendelian randomization.

### Microbiota causally linked to cholelithiasis may be associated with bile salt hydrolase

**Figure 3:**
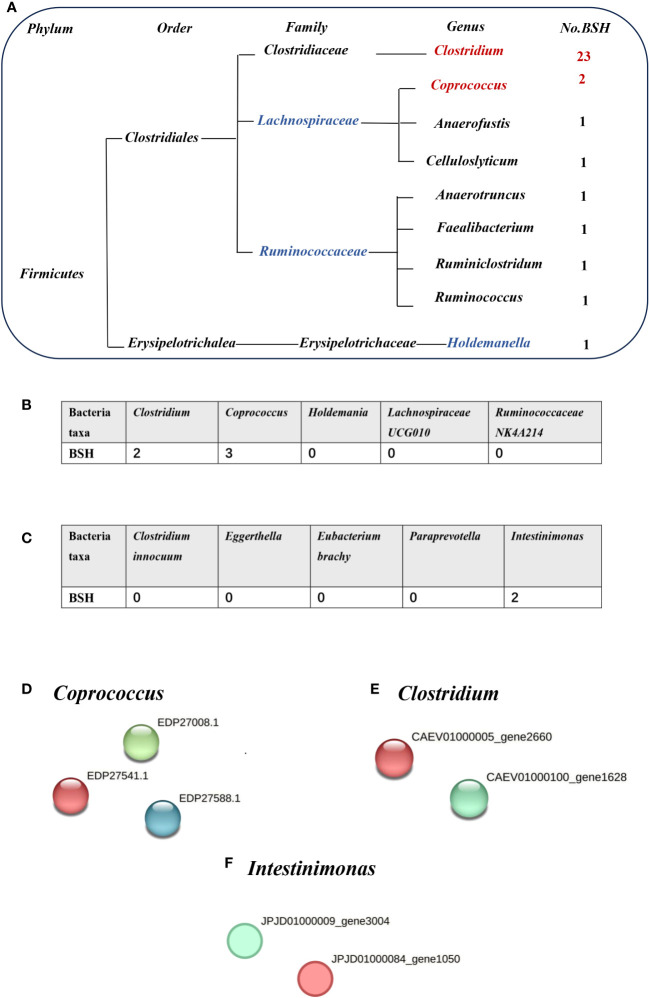
The number of bile salt hydrolases (BSHs) in microbiota. **(A)** The number of BSHs in microbiota genera base on HMP database. **(B)** The number of BSHs in microbiota genera based on Mendelian randomization (MR) analysis and reverse MR analysis **(C)**. The number of BSH- related proteins in **(D)** *Coprococcus*, **(E)** *Clostridium*, **(F)** *Intestinimonas*, red represents BSH, blue and green dots represent BSH-related proteins.

**Figure 4:**
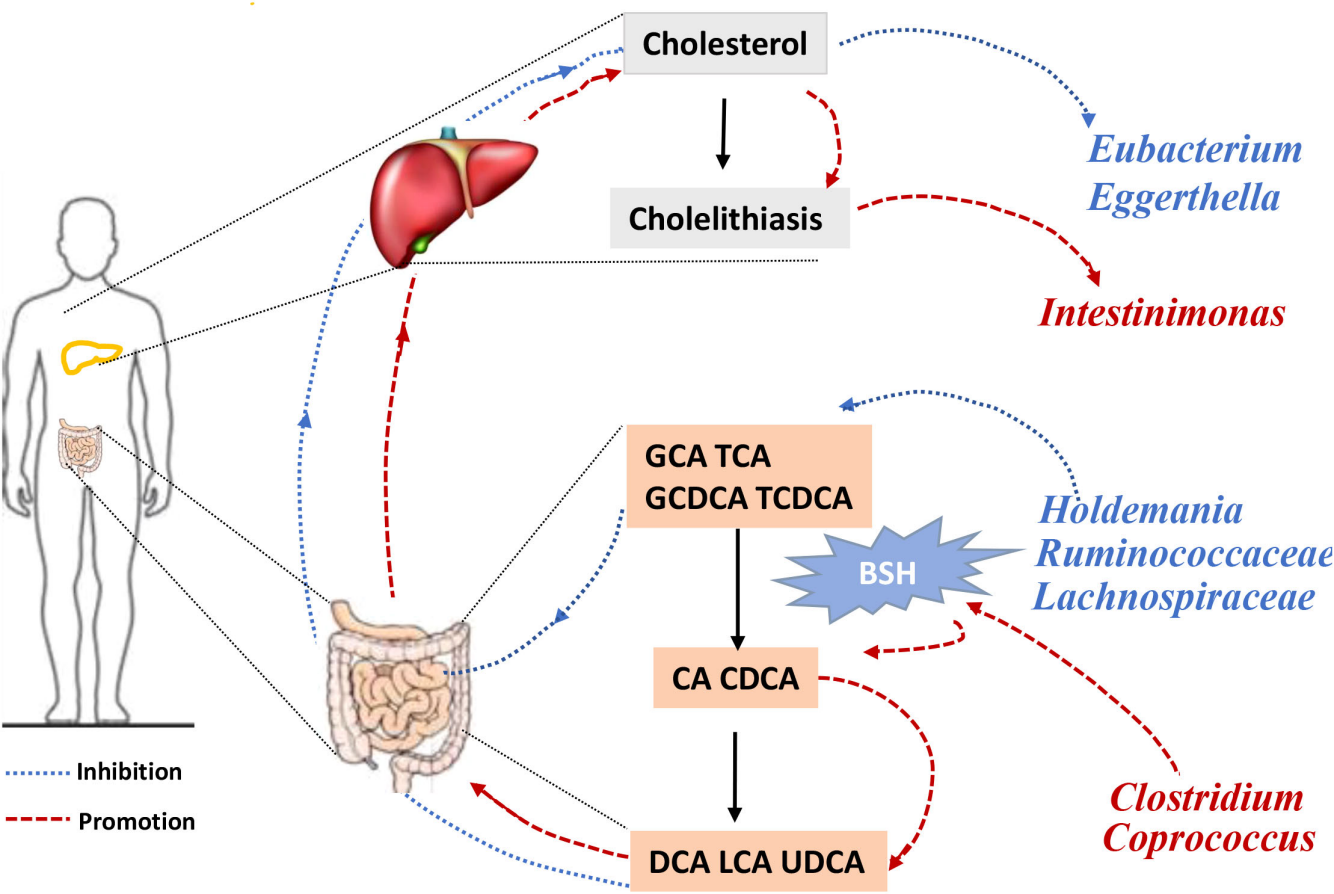
The framework of two-sample Mendelian randomization analysis results. Gut microbiota causally influences cholelithiasis, while cholelithiasis impacts the composition of gut microbiota. GCA, glycocholic acid; TCA, taurocholic acid. GCDCA, Glycochenodeoxycholic acid; TCDCA, Taurochnodeoxycholic acid; CA, Cholic acid; CDCA, Chenodeoxycholic acid; DCA, Deoxycholic acid; LCA, Lithocholic acid; UDCA, Ursodeoxycholic acid; BSH, bile salt hydrolase.

Bile acids (BAs) are divided into primary and secondary categories. Primary BAs are excreted into the intestine and converted into secondary BAs by intestinal microorganisms (Sinha et al., 2020). The initial step involves hydrolysis of the amino acid fraction by BSH during secondary BA metabolism (Smirnova et al., 2022). BSH (also known as choloylglycine hydrolase) is present in the intestinal microbiome to maintain BA balance. Imbalances in BAs are associated with gallstones, gallbladder disease, obesity, and diabetes (Cai et al., 2022a). BSHs are highly conserved in all major gut microbial phyla (including Bacteroidetes, Firmicutes, and Actinobacteria); however, they are bacterially different owing to their preferential activity toward glycine- or taurine-conjugated BAs. The Human Microbiome Project reported that 26.03% bacterial strains encode BSHs (Song et al., 2019). Thus, gut microbiota-related BSH directly determines the synthesis of secondary bile acids, which involve in regulating cholesterol metabolism. We hypothesized that BSH may be a link between intestinal microbiota and gallstone causation. Thus, we selected BSHs of the microbiome (that are associated with cholelithiasis by MR analysis) in the Human Microbiome Project database and National Center for Biotechnology Information database for protein–protein interaction (PPI) analysis. *Clostridium* and *Coprococcus* were predicted as a risk factor for cholelithiasis by MR and they expressed more BSHs than the protective bacteria (*Holdemania*, *Lachnospiraceae UCG010*, and *Ruminococcaceae NK4A214*) (**Figures 3A, B, D, E**). Similarly, inverse MR analysis predicted cholelithiasis as a risk factor for *Intestinimonas*, which expresses BSH (**Figures 3C, F**). We also used STRING to predicted the function of Clostridium, Coprococcus and *Intestinimonas*. Enrichment analysis showed that the bile acid catabolic process and secondary bile acid biosynthesis were enriched in *Clostridium* and *Coprococcus* according to Gene Ontology analysis and Kyoto Encyclopedia of Genes and Genomes pathway analysis*. Intestinimonas* was only enriched for secondary bile acid biosynthesis (**Table S2**). No enrichment of secondary bile acid biosynthesis in microbiota was observed that did not contain the BSH protein. These results suggest that BSH may serve as a link between gut microbiota and cholelithiasis (**Figure 4**).

### Microbiota linked to serum total cholesterol may be associated with BSH

**Figure 5:**
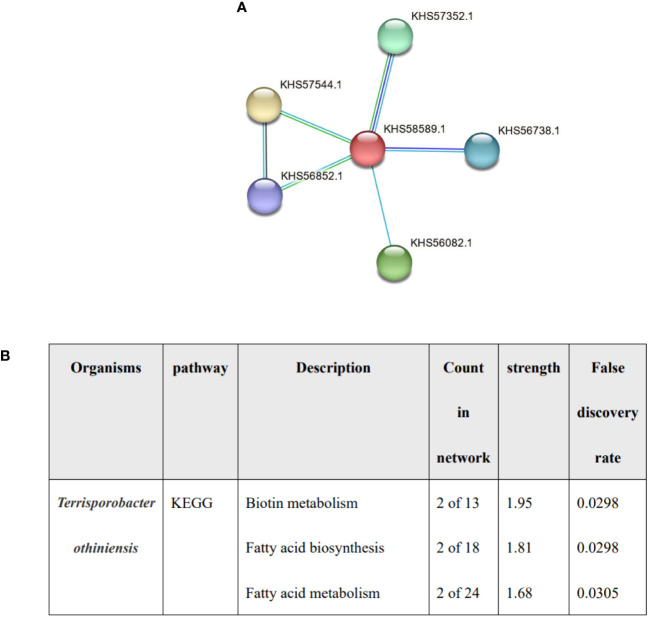
The analysis of Terrisporobacter othiniensis by protein-protein interaction (PPI). **(A)** The number of BSHs in Terrisporobacter othiniensis, red represents BSH. **(B)** Functional prediction of Terrisporobacter othiniensis by STRING. BSH, bile salt hydrolase; KEGG, Kyoto Encyclopedia of Genes and Genomes; PPI, protein-protein interaction.

Lower total cholesterol levels in serum may be an independent risk factor for cholelithiasis (Chen et al., 2022). It has been proved that one of the effects of BSH on host is cholesterol lowering (Ertürkmen et al., 2023), and *Terrisporobacter* was associated with higher total cholesterol levels (Guo et al., 2023). We detected the expression of HBS in *Terrisporobacter*, the red node represented BSH, and the functional enrichments was predicted by STRING, no enrichments were found (PPI enrichment P value: 0.457) and the function was described protein lipoylation and CoA hydrolase activity rather than bile acid or fatty acid metabolism (**Figure 5**). the result indicated that BSH of *Terrisporobacter* may be little function in bile acid or fatty acid metabolism.

## Discussion

This study used the MiBioGen database and cholelithiasis data from FinnGen to investigate the causal relationship between gut microbes and cholelithiasis using MR analysis. *Clostridium senegalense*, *Coprococcus3*, and *Lentisphaerae* increase the risk of cholelithiasis. In contrast, *Holdemania*, *Lachnospiraceae* UCG010, and *Ruminococcaceae* NK4A214 showed protective effects. The causal relationship identified BSH as a potential link between bile salt metabolism and the gut microbiota.

The intestinal microbiota is a metabolic organ that produces numerous metabolites (Connell et al., 2022) (including BAs and indole derivatives) (Agus et al., 2018) that play crucial roles in regulating host metabolism (Cai et al., 2022b). Cholesterol oxidized by liver enzymes results in the production of BAs that are further metabolized by the intestinal microbiota (Collins et al., 2023). The gut microbiota regulates key enzymes, such as cholesterol-7α-hydroxylase (CYP7A1), involved in BA synthesis (Hartmann et al., 2018; Fukui, 2021), and BA synthesis is tightly controlled by negative feedback inhibition through the farnesoid X receptor (FXR) (Jia et al., 2018). Bile acid deconjugation is primarily mediated by bacteria with BSH activity (Smirnova et al., 2022). Thus, intestinal microbiota plays a significant role in the key enzymatic processes involved in cholesterol synthesis in the liver, secondary BA production, and enterohepatic circulation of BAs.

The balance between cholesterol and bile salts is a critical factor for gallstones (Ye et al., 2021). Previous studies have revealed direct associations between taurocholic acid, taurochenodeoxycholic acid, and alpha diversity of the microbiota, together with positive associations with the genera *Chitinophagaceae*, *Microbacterium*, *Lutibacterium*, and *Prevotella intermedia* (Petrov et al., 2020). Patients with gallstones exhibit an increased richness of 7a-dehydroxylating microbiota and decreased levels of *Fimicutes and diversity of gut microbiota* (Wang et al., 2020). Additionally, the abundance of *Lactobacillus* strains significantly reduced in lithogenic diet-induced gallstones through the mediation of FXR signaling (Ye et al., 2022). However, the causal correlation between the intestinal flora and cholelithiasis remains unclear. Our study predicted that presence of abundance of *Clostridium senegalense* and *Coprococcus3* have a causal relationship with cholelithiasis.

Many observational studies report a correlation between *Clostridium* and gallstone disease. For instance, patients with gallstones have elevated levels of *Clostridium* in the stool (Grigor’eva and Romanova, 2020), and *Clostridium* species were isolated from gallbladder stones (Liu et al., 2000), consistent with the results of our study. We found a causal association between *Clostridium senegalense* and cholelithiasis. *Clostridium*-encoded protein analysis revealed that *Clostridium* had two genes of BSH consistent with a previous study (Song et al., 2019). The presence of greater amounts of bacteria with BSH leads to increased bile salt deconjugation, resulting in elevated biliary deoxycholate levels, positive regulation of hepatic cholesterol secretion, and cholesterol crystallization (Lammert et al., 2016). Furthermore, increased hydrolysis can lead to steatosis, and increased secondary bile acid levels are associated with colorectal cancer (Horáčková et al., 2018). Recent research showing that theabrownin can reduce hypercholesterolemia by inhibiting the intestinal microbiota related to BSH activity indirectly supports our hypothesis. The underlying mechanism may be that low BSH activity increased the concentration of conjugated BAs, which could inhibit the FXR-FGF15 signaling pathway, thereby led to decreased hepatic cholesterol synthesis (Huang et al., 2019). However, the effect of BSH on the microbiota in cholelithiasis causation requires verification and further exploration.

*Coprococcus* was also predicted to be a risk factor for gallstone disease, and encoded BSH proteins which was similar to the previous study (Song et al., 2019). Moreover, the relative abundance of BSH in the microbiota is significantly associated with mortality from diabetes and cardiovascular disease (Song et al., 2019). This finding further emphasizes the significance of BSH in cholelithiasis. Further research is required to understand how BSH in *Clostridium* and *Coprococcus* affect the formation of cholesterol stones. Recent studies show that *Desulfovibrionales* are enriched in cholelithiasis patients, increase the synthesis of secondary BAs and intestinal cholesterol uptake, stimulate biliary secretion, and affect FXR and CYP7A expression [15]. Further research is required to determine whether BSHs in *Clostridium* and *Coprococcus* promote cholesterol stone formation by increasing the secondary BA synthesis.

The present study revealed the protective effects of *Lachnospiraceae UCG010* and *Ruminococcaceae NK4A214* against cholelithiasis. These strains did not express BSH. This result suggests that BSH deficiency might be a protective factor against gallstone formation; however, this hypothesis requires verification. Microbial conversion of cholesterol to coprostanol is another mechanism that reduces cholesterol and decreases the formation of cholesterol stones (Kenny et al., 2020). *Lactobacillus curvatus KFP419* strain reduce cholesterol levels by increasing the conversion of cholesterol to coprostanol (Park et al., 2018). *Lachnospiraceae* and *Ruminococcaceae* are reportedly associated with high coprostanol levels (Antharam et al., 2016). An elevation of sterol metabolites coincides with increases in the *Lachnospiraceae* family *in vitro*, implying that this family promotes sterol metabolism (Blanco-Morales et al., 2020). These findings suggest that the protective effects of *Lachnospiraceae* and *Ruminococcaceae* against cholelithiasis may be related to cholesterol conversion; however, the underlying mechanisms require further exploration.

To the best of our knowledge, this is the first MR investigation revealing a link between gut microbiota and cholelithiasis. Robust genetic instruments were employed with a test of horizontal pleiotropy and two-sample heterogeneity to obtain reliable results from the MR analysis. Additionally, a leave-one-out analysis was performed to examine potential biases introduced by individual SNPs. Five sets of genetic instruments were used for MR analysis. In addition, the concept of BSH was introduced as an innovative approach to investigate the underlying mechanisms of the causal association between microbiota and cholelithiasis.

Nevertheless, this study has limitations. First, most patients with cholelithiasis in the analysis were of European descent, whereas the gut flora database encompasses other populations. Second, the potential association between microbiota and cholelithiasis by BSH was only superficially speculated. Further research is required to elucidate the underlying mechanisms and causal relationship. Third, subgroup analyses such as distinguishing between symptomatic and asymptomatic cholelithiasis was not possible because summary data for cholelithiasis was used in our analysis. Fourth, our exploration was limited to the genus level owing to the lowest taxonomic level available in the gut microbiota dataset, thus impeding a more detailed investigation at the species level. Additionally, some bacteria were predicted to be present at the phylum level; we could not analyze their BSH proteins. Finally, the sample size of the exposure group was relatively small; therefore, the reverse MR analysis results could not completely exclude the possibility of reverse causality.

## Conclusion

Our investigations demonstrated a causal relationship between cholelithiasis and *Clostridium senegalense* and *Coprococcus3*, whereas *Holdemania*, *Lachnospiraceae UCG010*, and *Ruminococcaceae NK4A214* had a protective effect. The causal relationship between the gut microbiota and cholelithiasis may be mediated by BSH. In addition, reverse MR analysis supported a causal relationship between cholelithiasis and the intestinal microbiota. Moreover, these findings suggest that cholelithiasis may influence the gut flora. Further validation and mechanistic studies are required.

## Data availability statement

The datasets presented in this study can be found in online repositories. The names of the repository/repositories and accession number(s) can be found in the article/**Supplementary Material**.

## Ethics statement

Ethical approval was not required for the study involving humans in accordance with the local legislation and institutional requirements. Written informed consent to participate in this study was not required from the participants or the participants’ legal guardians/next of kin in accordance with the national legislation and the institutional requirements.

## Author contributions

XL: Data curation, Formal Analysis, Investigation, Methodology, Resources, Visualization, Writing – original draft, Writing – review & editing. XQ: Data curation, Formal Analysis, Methodology, Writing – review & editing. RH: Data curation, Validation, Writing – original draft. TM: Supervision, Writing – review & editing. ZT: Funding acquisition, Investigation, Project administration, Writing – review & editing.
