# Supplementary material for: Gut microbiota causally affects cholelithiasis: a two-sample Mendelian randomization study
Source: Front Cell Infect Microbiol. 2023 Oct 9;13:1253447. doi: 10.3389/fcimb.2023.1253447 (PMC10591199; doi:10.3389/fcimb.2023.1253447)
Supplement: Supplementary file 1 [file Table_1.docx]

**Table S1** Sensitivity analysis

| Exposure | Cochran’s Q | MRegger_  interpreter | MRegger interpreter pval | MRPRESSO  -global |
| --- | --- | --- | --- | --- |
| Genus *Clostridium sensustricto1* | 0.80 | -0.0197 | 0.49 | 0.83 |
| Genus *Coprococcus3* | 0.73 | 0.0210 | 0.65 | 0.76 |
| *Genus Holdemania* | 0.07 | 0.0205 | 0.42 | 0.09 |
| Genus *Lachnospiraceae* UCG010 | 0.18 | 0.0099 | 0.74 | 0.19 |
| Genus *Ruminococcaceae* NK4A214 | 0.27 | -0.0006 | 0.98 | 0.29 |
| Phylum *Lentisphaerae* | 0.12 | -0.0153 | 0.73 | 0.13 |

**Table S2** Functional prediction of microbiota by STRING.

| **Organisms** | **pathway** | **Description** | **Count**  **in**  **network** | **strength** | **False discovery rate** |
| --- | --- | --- | --- | --- | --- |
| ***Coprococcus*** | GO-term | Bile acid catabolic process | 3 of 3 | 3.0 | 5.24e-06 |
|  | KEGG | Secondary bile acid biosynthesis | 2 of 2 | 3.0 | 0.00042 |
| ***Clostridium*** | GO-term | Bile acid catabolic process | 2 of 2 | 3.28 | 0.001 |
|  | KEGG | Secondary bile acid biosynthesis | 2 of 2 | 3.28 | 9.14e-05 |
| ***Intestinimonas*** | GO-term | Bile acid catabolic process | 2 of 2 | 3.21 | 0.0014 |

Abbreviations: GO, gene ontology; KEGG, Kyoto Encyclopedia of Genes and Genomes
